# Self-efficacy, motivation, and habits: psychological correlates of exercise among women with breast cancer

**DOI:** 10.1007/s00520-023-08040-7

**Published:** 2023-09-20

**Authors:** Tamara L. Jones, Lara Edbrooke, Jonathan C. Rawstorn, Sandra C. Hayes, Ralph Maddison, Linda Denehy, Camille E. Short

**Affiliations:** 1 Melbourne School of Psychological Sciences, The University of Melbourne, Melbourne, Australia; 2 Melbourne School of Health Sciences, The University of Melbourne, Melbourne, Australia; 3 Department of Health Services Research, The Peter MacCallum Cancer Centre, Melbourne, Australia; 4 Institute for Physical Activity and Nutrition, Deakin University, Melbourne, Australia; 5 Menzies Health Institute Queensland, Griffith University, Brisbane, Australia; 6 School of Health Sciences and Social Work, Griffith University, Brisbane, Australia

**Keywords:** Breast cancer, Exercise, Theory integration, Behaviour change

## Abstract

**Purpose:**

The purpose of this analysis was to explore associations between exercise behaviour among breast cancer survivors and three behavioural constructs from distinct theories: self-efficacy from social cognitive theory, motivation from self-determination theory, and habits from habit theory.

**Methods:**

Breast cancer survivors (*n* = 204) completed a cross-sectional survey that collected demographic and disease characteristics, exercise levels, and self-efficacy, motivation, and habits. Multivariable linear regression models were used to identify constructs associated with total activity and resistance training.

**Results:**

Participants were a mean (SD) age of 57.3 (10.8) years and most were diagnosed with early-stage disease (72%) and engaged in sufficient levels of total activity (94%), though only 45% completed ≥ 2 resistance training sessions/week. Identified motivation (*ꞵ*[95% CI] = 7.6 [3.9–11.3]) and habits (*ꞵ*[95% CI] = 4.4 [1.4–7.4]) were significantly associated with total activity (as were body mass index and disease stage), whilst identified motivation (*ꞵ*[95% CI] = 0.6 [0.3–0.9]) and coping self-efficacy (*ꞵ*[95% CI] = 0.02 [< 0.01–0.03]) were significantly associated with resistance training. The models explained 27% and 16% of variance in total activity and resistance training behaviour, respectively.

**Conclusion:**

Results suggest that incorporating strategies that support identified motivation, habits, and coping self-efficacy in future interventions could promote increased exercise behaviour among breast cancer populations. Future longitudinal research should examine associations with exercise in a more representative, population-based sample.

**Supplementary Information:**

The online version contains supplementary material available at 10.1007/s00520-023-08040-7.

## Introduction

Detection and treatment of breast cancer has drastically improved in recent decades, and breast cancer survivors now have one of the highest rates of cancer survival [1, 2]. However, breast cancer survivors can experience debilitating physical and psychological effects resulting from treatment, with up to 90% reporting persistent treatment-related sequelae [3, 4]. As such, strategies designed to enhance the quality of breast cancer survivorship have received considerable research attention [5]. Exercise has been identified as one such strategy, and the role of regular exercise in surviving breast cancer well is internationally recognised [6, 7].

Exercise is a subset of physical activity that is structured, planned, and conducted to improve physical and psychosocial outcomes [8]. A compelling body of interventional evidence supports the efficacy of exercise in the management of cancer-related health outcomes, including health-related quality-of-life, physical function, fatigue, anxiety, and depressive symptoms [6, 7]. As a result, several health organisations have published recommendations promoting the importance of regular exercise for people with cancer [6, 7]. Unfortunately, observational evidence indicates that physical activity levels (any bodily movement, including exercise) typically decline post-breast cancer diagnosis and remain low beyond the active treatment phase [9, 10].

To support breast cancer survivors to adopt and maintain regular exercise, effective behaviour change support programs are needed. In order to design such programs, an understanding of characteristics that influence exercise behaviour is required. Psychological factors are of particular interest, as they are considered to be malleable through behaviour change intervention and account for a reasonable variance (26–48%) in an individual’s behaviour [11–13]. To date, social cognitive models of behaviour have been the predominant framework for understanding (and promoting) exercise behaviour among breast cancer survivors, with self-efficacy a key focus [14]. Review level evidence supports the value of focusing on self-efficacy, with positive associations between self-efficacy and changes in physical activity and exercise consistently observed among cancer patients [15].

Generally, social cognitive models suggest that people make intentions or goals based on the deliberation of their beliefs (including self-efficacy beliefs) and that behaviour change occurs through the translation of beliefs into actions [16]. To aid this process, interventions based on social cognitive theories tend to focus on education and purposeful planned action, commonly including techniques such as goal setting, action planning, and self-monitoring [14, 17, 18]. Overall, interventions employing these techniques among breast cancer survivors have been promising, with small-modest improvements observed in the short and medium term [14, 17, 18]. Specifically, a study exploring social cognitive theory and breast cancer reported that self-efficacy, goals, social support, fatigue (a barrier), and outcome expectations, explained 41–49% of variance [19]. However, further gains may be achieved by drawing on other theoretical frameworks.

One concept that may be particularly useful to consider is the motivation continuum from self-determination theory, which views motivation as a continuum of self-determination [11, 20]. More self-determined forms of motivation (identified and intrinsic regulation) have been associated with meeting physical activity guidelines [21]. In particular, identified regulation (valuing physical activity as personally important) appears to play an important role in short-term uptake, whilst intrinsic motivation (being active for inherent enjoyment or satisfaction) appears to be more important for promoting behaviour change maintenance [21]. Intrinsic motivation is supported when people meet their basic psychological needs of relatedness (meaningful connections with others), autonomy (a sense of choice, control over one’s behaviour), and competence (feeling capable and effective when completing a task, similar to task self-efficacy) [11, 22]. Among breast cancer survivors, a study exploring social determination theory found that 20% of variance in exercise behaviour was explained by identified regulation and competence [21].

Habit formation from habit theory [23] may also lead to longer-term behaviour changes, as repeating actions consistently in the same context creates semi-automated behaviours that become less dependent on attention and motivational processes [24]. Habits have consistently been associated with exercise behaviour in the general population (even when controlling for motivation [24]). Further, intervention research has shown strategies to promote habits within standard social cognitive approaches (e.g., encouraging behavioural consistency when forming action plans) result in greater exercise maintenance than when these strategies aren’t used [25]. However, the impact of habits or promoting habit formation in conjunction with other behavioural techniques in a breast cancer population has not been well investigated.

While individual theories have been explored among breast cancer survivors, there is limited evidence available exploring the potentially increased benefits that may arise from being guided by integrated theoretical frameworks. However, previous work conducted in both clinical and healthy populations suggests that greater variance may be explained by integrating behaviour change constructs from various theories [20, 26]. Through theory integration, researchers can use complimentary constructs to address the limitations of individual theories [11] and may be able to provide a more thorough explanation of exercise behaviour [11]. This research aims to inform such work by exploring the association between exercise behaviour and distinct psychological constructs from three promising theoretical perspectives among women with breast cancer, namely, the association between exercise and self-efficacy, motivation, and habits.

## Materials and methods

This cross-sectional study involved the completion of an online survey between March and December, 2020. Ethics approval was obtained from the University of Melbourne Human Research Ethics Committee (#1955472).

### Participants and procedure

Participants were recruited via paid Facebook advertisements (March and June, 2020) and cancer specific research registries (National Breast Cancer Foundation Register4 [December 2020], Breast Cancer Network Australia membership list [July 2020]). Eligible participants were those ≥ 18 years and diagnosed with breast cancer in the past 5 years; able to answer the survey in English; and residing in Australia. The survey was conducted using the Qualtrics platform. All potentially eligible participants were directed to the online survey where they could read the study information sheet, provide informed consent, and complete the survey. Upon completion of the survey, participants could leave contact details if they wished to receive a $5 gift card for completing the survey, receive a summary of the results, and/or be contacted for future research opportunities.

### Survey

Survey items were informed and reviewed by two breast cancer consumer representatives and the multidisciplinary research team (physiotherapists, behavioural scientists, a dietician, cardiologist, and medical oncologist). Survey items collected information regarding demographic characteristics and medical history, types of exercise support previously provided, preferences for exercise programs and likelihood of telerehabilitation uptake, current exercise and nutrition behaviours, and psychological determinants of exercise. Assessment methods for the outcomes of interest in the current analysis are described below.

#### Demographic and disease characteristics

Participants self-reported age, postcode (used to determine degree of remoteness based on Australian Statistical Geography Standard, [27]), marital status, education, employment, height, weight, and previous diagnosis of other health conditions. A study-specific comorbidity index was created by summing reported health conditions (range = 0–8, including high cholesterol, high blood pressure, diabetes, congestive heart failure, heart attack, stroke or transient ischaemic attack, depression and/or anxiety, or other). Disease characteristics including stage at diagnosis, treatments received, time since diagnosis, and current disease state were also self-reported.

#### Psychological constructs

Self-efficacy (Multidimensional Self-efficacy for Exercise Scale [MSES] [28]), motivation (Behavioral Regulation in Exercise Questionnaire Version 2 [BREQ-2] [29]), and habits (Self-Report Habit Index [SRHI] [30]) were assessed using validated self-report questionnaires. The MSES is a 9-item tool that assesses task (confidence performing elemental aspects of exercise), coping (confidence exercising under challenging circumstances), and scheduling (confidence exercising regularly despite other time demands) self-efficacy in relation to exercise behaviour. Each self-efficacy subtype is scored from 0 to 100, with higher scores indicating higher levels of self-efficacy. The BREQ-2 is a 19-item questionnaire that assesses exercise motivation across the self-determination continuum, including external, identified, introjected, and intrinsic regulations of exercise behaviour, and amotivation [29]. Each motivation type is score from 0 to 4, with higher scores indicating higher levels of that motivation type. The SRHI is a 4-item tool (scored from 1 to 5) that assesses the automaticity of a habitual behaviour (i.e., exercise) [30], with a higher score indicating greater automaticity in exercise behaviour.

#### Exercise

Exercise behaviour was assessed using the Godin Leisure Time Exercise Questionnaire (GLTEQ) [31] and a purpose-built resistance-training item using the same format. For the GLTEQ, participants indicated how many times they undertake strenuous (e.g., running), moderate (e.g., fast walking), and mild/light (e.g., easy walking) exercise for > 15 min during a typical 7-day period. Activity frequencies were multiplied by 9, 5, and 3 metabolic equivalents, respectively, and summed to create a total activity score. Participants were considered sufficiently active if their GLTEQ total activity score was ≥ 24 [32]. For resistance-training, participants indicated how many times they spent doing resistance training (strength) activities using the same question stem (participants completing ≥ 2 session per week were categorised as meeting guidelines) [6, 7]. Although resistance training may also be reported as a subset of exercise behaviour through the GLTEQ, a separate question was included to specifically collect resistance training information, as it is plausible that different psychological constructs may be associated with different exercise modes.

### Statistical analyses

Descriptive statistics were calculated for demographic and disease characteristics, psychological constructs, and exercise behaviour. Two backward stepwise multivariable linear regression models were constructed to identify possible psychological correlates of (1) total activity and (2) resistance training. Initially, bivariate linear regression models were used to examine the associations between individual characteristics (demographic, disease, and psychological determinants) and the outcome variables. Acknowledging the potential importance of demographics and disease characteristics in explaining health behaviours, bivariate analyses were conducted for all collected independent variables [33]. Variables that were statistically significant (*p* value ≤ 0.05) or clinically relevant (≥ 0.5SD of baseline exercise level) in the bivariate analyses were selected for inclusion in the multivariable models, in addition to variables of theoretical importance (i.e., age) [34]. Potential for multicollinearity was screened by examining associations between the remaining variables using correlation coefficients matrices. Where variables were found to be highly collinear (> 0.5) [35], the strongest variable (based on model coefficients) was retained. At each step, the variable with the highest *p* value was removed from the model (assuming it satisfied the elimination criterion, i.e., a *p* value > 0.05), unless it was clinically relevant, in which case it was retained. The procedure ceased when there were no variables in the model that satisfied the elimination criterion (i.e., all variables in the model were statistically significant or clinically relevant). All analyses were conducted on a complete case basis using SPSS Statistics for Windows, version 28.0 (SPSS Inc., Chicago, IL, USA).

## Results

### Participants

A total of 250 individuals provided informed consent and participated in the survey. Of those, 86% (*n* = 215) completed questions relating to the two outcomes of interest (i.e., total activity and resistance training) and 82% (*n* = 204) completed questions relating to all independent variables (i.e., demographic characteristics and psychological constructs).

Demographic and disease characteristics and exercise levels are reported in Table 1. Participants were diagnosed at a mean (SD) age of 57 (11) years and had a BMI of 27 (6). The majority resided in a major city (64%), were married (73%), and had attended university (66%). In terms of disease characteristics, 72% were diagnosed with early-stage disease and 57% had completed curative treatment.

**Table 1:** Demographic, disease, and exercise characteristics of complete cases (*n* = 204)

	Mean (SD)	Median (range)
Demographic characteristics		
Age, years	57.3 (10.8)	58.0 (24.0–84.0)
BMI, kg/m^2^	27.2 (5.7)	26.0 (18.2–56.9)
Comorbidity index	0.9 (1.0)	1 (0.0–5.0)
Location	***N* (%)**	
Major cities Australia	130 (63.7)	
Inner regional/outer regional/remote Australia	74 (36.3)	
Marital status	***N* (%)**	
Married, de facto, or living with partner	149 (73.0)	
Separated, divorced, widowed, single	53 (26.0)	
Prefer not to say	2 (1.0)	
Education	***N* (%)**	
High school (year 10 or 12)	16 (7.8)	
Certificate or diploma (e.g., TAFE or College)	53 (26.0)	
University degree	135 (66.2)	
Employment	***N* (%)**	
Employed	102 (50.0)	
Retired	64 (31.4)	
Other	38 (18.6)	
Disease characteristics		
Time since diagnosis, years	2.2 (1.6)	1.9 (0.1–5.1)
Stage of disease^a^	***N* (%)**	
Stages I–II	146 (71.6)	
Stages III–IV	55 (27.0)	
Unsure	3 (1.5)	
Treatment types	***N* (%)**	
Surgery	196 (96.1)	
Radiation therapy	152 (74.5)	
Chemotherapy	111 (54.4)	
Hormone therapies	120 (58.8)	
Trastuzuman	27 (13.2)	
Immunotherapy	9 (4.4)	
Other	8 (3.9)	
Treatment stage	**N (%)**	
Not yet started active treatment for cancer	2 (1.0)	
Current undergoing curative treatment	37 (18.1)	
Completed curative treatment and in remission	117 (57.4)	
Ongoing treatment to manage the disease	45 (22.1)	
Other	3 (1.5)	
Exercise levels		
GLTEQ total activity score, units	49.4 (20.6)	45.5 (17.0–127.0)
Resistance training, sessions per week	2.6 (1.7)	2.0 (1.0–8.0)
GLTEQ physical activity categories^b^	***N* (%)**	
Moderately active (total activity score < 24)	13 (6.4)	
Active (total activity score ≥ 24)	191 (93.6)	
Resistance training categories	***N* (%)**	
Not meeting guidelines (< 2 sessions/week)	113 (55.4)	
Meeting guidelines (≥ 2 sessions/week)	91 (44.6)	

^a^Self-reported as stage I or II (early stage), stage III (locally advanced), stage IV (advanced or metastatic), or unsure [36]

^b^No participants were classified as sedentary (total activity score = 0)

Most participants (94%) were regularly engaging in recommended levels of exercise (i.e., GLTEQ total activity score of ≥ 24), with no participants classified as ‘Sedentary’. Despite this, less than half the sample (45%) was meeting resistance training guidelines (i.e., two sessions per week). Total activity levels were significantly lower among participants who had missing data for independent variables (*n* = 11) than for those with complete data (36.1 (15.4) vs. 49.4 (20.6); *p* = 0.04).

Psychological determinant scores of the sample are presented in Table 2. High scores were commonly reported for both task and scheduling self-efficacy. High scores were also commonly reported for motivation types at the higher end of the self-determination continuum (identified and intrinsic). Lower scores were reported for external and amotivation.

**Table 2:** Psychological characteristics and bivariate associations with exercise (*n* = 204)

	Descriptive results		Total activity (GLTEQ total activity score)	Resistance training (number of sessions per week)
Psychological constructs	Mean (SD)	Median (range)	*ꞵ* (95% CI)	*ꞵ* (95% CI)
Self-efficacy				
Task self-efficacy (0–100)	74.1 (19.3)	78.7 (6.0–100.0)	0.19 (0.04, 0.33)*	0.03 (0.01, 0.04)*
Coping self-efficacy (0–100)	51.4 (20.6)	51.7 (7.7–96.3)	0.24 (0.10. 0.37)*	0.02 (0.01, 0.04)*
Scheduling self-efficacy (0–100)	75.2 (24.5)	81.8 (4.7–100.0)	0.37 (0.27, 0.47)*	0.03 (0.02, 0.04)*
Motivation				
Amotivation (0–4)	0.2 (0.5)	0.0 (0.0–3.0)	− 10.62 (− 16.58, − 4.65)*	− 0.52 (− 1.02, − 0.02)*
External motivation (0–4)	0.5 (0.7)	0.3 (0.0–4.0)	− 5.10 (− 9.19, − 1.02)*	− 0.19 (− 0.53, 0.15)
Introjected motivation (0–4)	1.6 (1.0)	1.7 (0.0–4.0)	− 2.23 (− 4.91, 0.45)	− 0.08 (− 0.30, 0.14)
Identified motivation (0–4)	3.2 (0.8)	3.5 (0.3–4.0)	11.38 (8.15, 14.61)*	0.76 (0.49, 1.04)*
Intrinsic motivation (0–4)	2.6 (1.1)	2.8 (0.0–4.0)	6.66 (4.20, 9.12)*	0.49 (0.29, 0.69)*
Habits				
Automaticity (1–5)	2.9 (1.0)	3.0 (1.0–5.0)	8.07 (5.32, 10.83)*	0.48 (0.24, 0.71)*

*Statistically significant: *p* < 0.05

### Bivariate analyses

Results of the bivariate analyses are reported in Table 2 (psychological constructs) and Supplementary Table 1 (demographic and disease characteristics). Independent variables that had a statistically significant bivariate association with total activity included BMI, education status, stage of disease, task, coping, and scheduling self-efficacy, amotivation, external, identified and intrinsic motivation, and habits (amotivation and identified motivation also had a clinically relevant association [≥ 0.5SD = 10.3] with total activity). Independent variables that had a statistically significant bivariate association with resistance training included education status, comorbidity index, task, coping, and scheduling self-efficacy, amotivation, identified and intrinsic motivation, and habits (no independent variables had a clinically relevant association [≥ 0.5SD = 0.85] with resistance training). Following screening for multicollinearity (see Supplementary Table 2), scheduling self-efficacy, amotivation, and intrinsic motivation were removed and not included in the multivariable models.

### Multivariable Models

Results from the multivariable linear regression models are presented in Table 3. In the total activity model, higher scores for identified motivation (*β* = 7.6) and habit strength (*β* = 4.4) were significantly associated with higher levels of exercise. In contrast, a higher BMI (*β* =  − 0.5) and more advanced stage disease at diagnosis (*β* =  − 7.6) were significantly associated with lower levels of exercise. The model explained 27% of the variability in exercise behaviour (adjusted *R*^2^ = 0.27). In the resistance training model, higher scores for coping self-efficacy (*β* = 0.02) and identified motivation (*β* = 0.61) were significantly associated a higher number of resistance training sessions per week. The model explained 16% of the variability in exercise behaviour (adjusted *R*^2^ = 0.16). There were no clinically relevant associations in either the total activity or resistance training models.

**Table 3:** Associations between demographic and disease characteristics, psychological constructs, and exercise behaviour (*n* = 204)

Characteristics	*ꞵ*	SE	95% Wald CI		*P*
			Lower	Upper	
Total activity (GLTEQ total activity score)					
Age	0.02	0.12	− 0.22	0.25	0.89
Body mass index	− 0.49	0.23	− 0.95	− 0.02	0.04
Stage of disease	− 7.57	2.78	− 13.04	− 2.10	0.01
Identified motivation	7.61	1.88	3.90	11.33	< 0.01
Habits	4.40	1.51	1.42	7.39	< 0.01
Resistance training (number of sessions per week)					
Age	− 0.01	0.01	− 0.03	0.01	0.29
Coping self-efficacy	0.02	0.01	< 0.01	0.03	< 0.01
Identified motivation	0. 61	0.15	0.32	0.90	< 0.01

## Discussion and conclusion

### Discussion

The present analysis demonstrates associations between exercise behaviour and psychological constructs from three theoretical perspectives. Identified motivation, habits, stage of disease, and BMI were significantly associated with total activity while identified motivation and coping self-efficacy were significantly associated with resistance training, explaining 27% and 16% of the variance in exercise behaviour respectively. These results are in line with previous findings exploring social cognitive and self-determination determinants, which explained 15% and 20% of variance, respectively [21, 37]. Further, these findings extend the literature by exploring a core construct from both social cognitive and self-determination theory in combination with habit theory, which lends support to the use of integrated theoretical frameworks for developing interventions to promote exercise among breast cancer survivors.

Social cognitive models of behaviour have been the predominant approach to promoting exercise in breast cancer survivors [16, 17] and often focus on strategies aimed at improving self-efficacy [14, 18]. However, in the current analysis, only coping self-efficacy (often referred to as barrier self-efficacy) retained a significant association with resistance training in the multivariable model. Compared to coping self-efficacy, participants reported higher scores for task and scheduling self-efficacy, so a ceiling effect may provide an explanation as to why these subtypes were not significantly associated with exercise behaviour in this sample. Further, generally high self-efficacy scores across all three subtypes may provide some explanation for why self-efficacy coefficients were smaller than those observed for motivation and habits. These results, in combination with the significance of identified motivation in both multivariable models, and habits in the total activity model, suggest that there is opportunity to influence exercise levels among people with breast cancer through an integrated theoretical approach. From this perspective, intervention developers could use evidence from self-determination theory and habit theory in conjunction with strategies to improve coping self-efficacy from social cognitive theory (such as self-monitoring and engaging support networks [38]) to design more effective intervention strategies to support exercise behaviour change.

Specifically, the results of this analysis highlight that more self-determined forms of motivation (i.e., identified motivation) are associated with more favourable exercise behaviours [11]. As such, incorporating strategies that enhance more intrinsic forms of motivation may be most useful for promoting exercise behaviour, which according to self-determination theory, could be achieved by helping people meet their basic psychological needs of relatedness, autonomy, and competence [11, 22]. Motivational interviewing techniques, which hinge on a positive interpersonal relationship and focus on evoking the person’s own reason for change could be a useful strategy for enhancing identified motivation [39]. Open questions are asked to help the person identify and articulate their intrinsic values and goals, and strengths and small successes are affirmed to help build confidence. In this way, relatedness, autonomy, and competence could all be addressed [39]. Techniques commonly employed in interventions guided by social cognitive theories (see Table 3 [14]) may also help to increase more self-determined motivation, given the focus on enhancing social support, self-efficacy, and outcome expectations. However, self-determination theory adds a valuable perspective in terms of promoting outcomes that are intrinsically regulated (such as enjoyment), rather than more externally regulated outcomes such as guilt [40]. Previous research in breast cancer patients has shown that those who report being motivated by feeling guilty when they skip exercising report 36% less exercise than those who are less motivated by that factor [40]. The focus on autonomy is also unique to self-determination theory and has implications for the structure of exercise programs. Autonomy could be supported by providing more choice regarding when and where to exercise, and/or what exercises to do. This should potentially be done alongside education on habit formation.

Habits have consistently been shown to be associated with exercise, even when controlling for motivation [23, 24]. As such, incorporating strategies that support the development of habits (e.g., providing habit formation advice) may be a valuable addition to interventions aimed at promoting sustained behaviour change. Habits are formed when a behaviour is performed automatically in response to a situational cue [24]. Previous literature supports strategies for habit formation that align with strategies supported by social cognitive and self-determination theory. Firstly, habits should be small and achievable to promote successful achievement and subsequent increases in self-efficacy and competences, which may encourage further behaviour change while avoiding feelings of failure [23]. Secondly, interventions should promote and support patients to choose the target behaviour that is personally important, rather than a behaviour that is externally motivated (e.g., physician recommendation). This approach will increase autonomy and allow patients to progress towards a self-determined goal, which in turn may contribute to higher intrinsic motivation and behaviour maintenance [41]. Although sufficient motivation is required to form a habit, once a habit has been established, performing that behaviour required less cognitive load (i.e., required minimal effort or deliberation) and is less dependent of motivation [23]. These habits are more likely to persist if other psychological correlates decrease [23].

Several limitations should be considered when drawing on the results. Firstly, the cross-sectional design does not allow us to draw conclusions about the causal nature of the associations or whether these constructs are correlated specifically with exercise adoption or maintenance*.* Further, the method of data analysis (i.e., backward stepwise multivariable linear regression) has some disadvantages including a reliance on statistical significance from single-step tests and an increased risk of overfitting data [42]. However, this analysis also considered the clinical relevance and theoretical importance of variables when selecting them for inclusion in the multivariable model. The study sample was also heterogeneous regarding treatment stage and highly skewed towards active women and those who are more highly educated. Although these are important factors to consider, the activity level of the sample is unsurprising considering the recruitment strategy (online advertising), as women who were already active or who valued exercise were more likely to respond to a survey regarding exercise and exercise preferences. An additional consideration is the self-report measure of physical activity, which tend to over-estimate physical activity levels when compared to objective assessment [43, 44]. Future research should aim to explore these associations in longitudinal studies (to examine correlates of exercise maintenance and the impact of specific treatment stages) and to test integrated theories involving all constructs. Additionally, these associations should be explored in a more representative, population-based sample. Specifically, among those who are less active, to determine if there are differences in the psychological constructs that play a role among those with differing levels of exercise or for specific exercise modes (i.e., resistance training). For this to occur, successful strategies to recruit less active participants will need to be developed, trialled, and refined. Embedding recruitment trials into future observational and experimental studies is recommended [45].

Despite these limitations, there are many strengths to the current analysis, such as the inclusion of psychological constructs that have yet to be extensively investigated in the breast cancer setting and the combination of psychological constructs from distinct theoretical frameworks. Despite being one of the most extensively investigated psychological constructs [15], most studies do not distinguish between self-efficacy types [46]. In the current analysis, three subtypes of self-efficacy (task, coping, and scheduling) were evaluated. Further, as well as examining correlates of total activity, this analysis also examined correlates of resistance training, which are less heavily researched in the literature [47]. The sample also included people from regional and remote geographic areas who are often not well represented in breast cancer research, with the proportion of participants residing outside of major cities (36%) being representative of the Australian population [48].

## Conclusion

Although preliminary, the findings of this study indicate that identified motivation, habits, and coping self-efficacy are associated with higher exercise levels among breast cancer survivors. Enhancing these constructs in future interventions could be a promising strategy to promote exercise in breast cancer populations. However, future longitudinal research should examine associations with exercise in a more representative, population-based sample.

### Supplementary Information

Below is the link to the electronic supplementary material.

Supplementary file1 (DOCX 60 KB)

## Data Availability

The datasets used and/or analysed during the current study are available from the corresponding author on reasonable request.

## References

[1] Australian Institute of Health and Welfare (2019) Cancer in Australia 2019. AIHW, Canberra. https://www.aihw.gov.au/reports/cancer/cancer-in-australia-2019/summary

[2] Surveillance Research Program, National Cancer Institute (2020) SEER*Explorer: an interactive website for SEER cancer statistics. https://seer.cancer.gov/explorer/

[3] Beckjord EB, Reynolds KA, van Londen GJ, Burns R, Singh R, Arvey SR, Nutt SA, Rechis R (2014). Population-level trends in posttreatment cancer survivors’ concerns and associated receipt of care: results from the 2006 and 2010 LIVESTRONG surveys. J Psychosoc Oncol.

[4] Lovelace DL, McDaniel LR, Golden D (2019). Long-term effects of breast cancer surgery, treatment, and survivor care. J Midwifery Womens Health.

[5] Vardy JL, Chan RJ, Koczwara B, Lisy K, Cohn RJ, Joske D, Dhillon HM, Jefford M (2019). Clinical oncology society of Australia position statement on cancer survivorship care. Aust J Gen Pract.

[6] Hayes S, Newton R, Spence R, Galvão D (2019). The exercise and sports science australia position statement: exercise medicine in cancer management. J Sci Med Sport.

[7] Campbell K, Winters-Stone K, Wiskemann J, May A, Schwartz A, Courneya K, Zucker D, Matthews C, Ligibel J, Gerber L, Morris GS, Patel A, Hue T, Perna F, Schmitz K (2019). Exercise guidelines for cancer survivors: consensus statement from international multidisciplinary roundtable. Med Sci Sports Exerc.

[8] Liguori G, Feito Y, Fountaine C, Roy B (2021). ACSM’s guidelines for exercise testing and prescription.

[9] De Groef A, Geraerts I, Demeyer H, Van der Gucht E, Dams L, de Kinkelder C, Dukers-van Althuis S, Van Kampen M, Devoogdt N (2018). Physical activity levels after treatment for breast cancer: two-year follow-up. The Breast.

[10] Mason C, Alfano CM, Smith AW, Wang C, Neuhouser ML, Duggan C, Bernstein L, Baumgartner KB, Baumgartner RN, Ballard-Barbash R, McTiernan A (2013). Long-term physical activity trends in breast cancer survivors cancer epidemiology. Biomarkers Prev.

[11] Hagger M, Chatzisarantis N (2008). Self-determination theory and the psychology of exercise. Int Rev Sport Exerc Psychol.

[12] Husebø AM, Dyrstad SM, Søreide JA, Bru E (2013). Predicting exercise adherence in cancer patients and survivors: a systematic review and meta-analysis of motivational and behavioural factors. J Clin Nurs.

[13] Young MD, Plotnikoff RC, Collins CE, Callister R, Morgan PJ (2014). Social cognitive theory and physical activity: a systematic review and meta-analysis. Obes Rev.

[14] Stacey FG, James EL, Chapman K, Courneya KS, Lubans DR (2015). A systematic review and meta-analysis of social cognitive theory-based physical activity and/or nutrition behavior change interventions for cancer survivors. J Cancer Surviv.

[15] Hoedjes M, Nijman I, Hinnen C (2022). Psychosocial determinants of lifestyle change after a cancer diagnosis: a systematic review of the literature. Cancers (Basel).

[16] Bandura A (2004). Health promotion by social cognitive means. Health Educ Behav.

[17] Short CE, James EL, Stacey F, Plotnikoff RC (2013). A qualitative synthesis of trials promoting physical activity behaviour change among post-treatment breast cancer survivors. J Cancer Surviv.

[18] Roberts AL, Fisher A, Smith L, Heinrich M, Potts HWW (2017). Digital health behaviour change interventions targeting physical activity and diet in cancer survivors: a systematic review and meta-analysis. J Cancer Surviv.

[19] Phillips SM, McAuley E (2013). Social cognitive influences on physical activity participation in long-term breast cancer survivors. Psychooncology.

[20] Sweet SN, Fortier MS, Strachan SM, Blanchard CM (2012). Testing and integrating self-determination theory and self-efficacy theory in a physical activity context. Can Psychol/Psychol Can.

[21] Milne HM, Wallman KE, Guilfoyle A, Gordon S, Corneya KS (2008). Self-determination theory and physical activity among breast cancer survivors. J Sport Exerc Psychol.

[22] Ryan RM, Deci EL (2000). Self-determination theory and the facilitation of intrinsic motivation, social development, and well-being. Am Psychol.

[23] Gardner B, Lally P, Wardle J (2012). Making health habitual: the psychology of ‘habit-formation’ and general practice. Br J Gen Pract.

[24] Rebar AL, Dimmock JA, Jackson B, Rhodes RE, Kates A, Starling J, Vandelanotte C (2016). A systematic review of the effects of non-conscious regulatory processes in physical activity. Health Psychol Rev.

[25] Kaushal N, Rhodes RE, Spence JC, Meldrum JT (2017). Increasing physical activity through principles of habit formation in new gym members: a randomized controlled trial. Ann Behav Med.

[26] Brooks JM, Iwanaga K, Chiu C-Y, Cotton BP, Deiches J, Morrison B, Moser E, Chan F (2017). Relationships between self-determination theory and theory of planned behavior applied to physical activity and exercise behavior in chronic pain Psychology. Health Med.

[27] Australian Bureau of Statistics (2005) Australian standard geographical classification (ASGC). Australian Bureau of Statistics, Canberra. https://www.abs.gov.au/AUSSTATS/abs@.nsf/Lookup/1216.0Main+Features12005?OpenDocument

[28] Rodgers WM, Wilson PM, Hall CR, Fraser SN, Murray TC (2008). Evidence for a multidimensional self-efficacy for exercise scale. Res Q Exerc Sport.

[29] Markland D, Tobin V (2004). A modification to the behavioural regulation in exercise questionnaire to include an assessment of amotivation. J Sport Exerc Psychol.

[30] Gardner B, Abraham C, Lally P, de Bruijn G-J (2012). Towards parsimony in habit measurement: testing the convergent and predictive validity of an automaticity subscale of the Self-Report Habit Index. Int J Behav Nutr Phys Act.

[31] Godin G, Shephard RJ (1985). A simple method to assess exercise behavior in the community. Can J Appl Sport Sci.

[32] Godin G (2011). The Godin-Shephard leisure-time physical activity questionnaire. Health Fitness J Canada.

[33] Kemp E, Trigg J, Beatty L, Christensen C, Dhillon HM, Maeder A, Williams PAH, Koczwara B (2021). Health literacy, digital health literacy and the implementation of digital health technologies in cancer care: the need for a strategic approach. Health Promot J Aust.

[34] Kampshoff CS, Jansen F, van Mechelen W, May AM, Brug J, Chinapaw MJM, Buffart LM (2014). Determinants of exercise adherence and maintenance among cancer survivors: a systematic review. Int J Behav Nutr Phys Act.

[35] Hosmer DW, Lemeshow S, Sturdivant RX (2013). Applied logistic regression.

[36] Cancer Australia (2021) Stages of breast cancer. Cancer Australia, Canberra. https://www.canceraustralia.gov.au/cancer-types/breast-cancer/symptoms-and-diagnosis/stages-breast-cancer

[37] Kampshoff CS, Stacey F, Short CE, van Mechelen W, Chinapaw MJM, Brug J, Plotnikoff R, James EL, Buffart LM (2016). Demographic, clinical, psychosocial, and environmental correlates of objectively assessed physical activity among breast cancer survivors. Support Care Cancer.

[38] Blanchard CM, Fortier M, Sweet S, O'Sullivan T, Hogg W, Reid RD, Sigal RJ (2007). Explaining physical activity levels from a self-efficacy perspective: the physical activity counseling trial. Ann Behav Med.

[39] Patrick H, Williams GC (2012). Self-determination theory: its application to health behavior and complementarity with motivational interviewing. Int J Behav Nutr Phys Act.

[40] Robertson MC, Liao Y, Song J, Lyons EJ, Basen-Engquist KM (2018). Motivation for physical activity and the moderating effect of cancer diagnosis: a nationally representative cross-sectional study. Prev Med.

[41] Gardner B, Lally P (2013). Does intrinsic motivation strengthen physical activity habit? Modeling relationships between self-determination, past behaviour, and habit strength. J Behav Med.

[42] Smith G (2018). Step away from stepwise. J Big Data.

[43] Douma JAJ, de Beaufort MB, Kampshoff CS, Persoon S, Vermaire JA, Chinapaw MJ, van Mechelen W, Nollet F, Kersten MJ, Smit JH, Verdonck-de Leeuw IM, Altenburg TM, Buffart LM (2020). Physical activity in patients with cancer: self-report versus accelerometer assessments. Support Care Cancer.

[44] Sallis JF, Saelens BE (2000). Assessment of physical activity by self-report: status, limitations, and future directions. Res Q Exerc Sport.

[45] Rick J, Graffy J, Knapp P, Small N, Collier DJ, Eldridge S, Kennedy A, Salisbury C, Treweek S, Torgerson D, Wallace P, Madurasinghe V, Hughes-Morley A, Bower P (2014) Systematic techniques for assisting recruitment to trials (START): study protocol for embedded, randomized controlled trials. Trials 15:407 10.1186/1745-6215-15-407 PMC4230578 25344684

[46] Higgins TJ, Middleton KR, Winner L, Janelle CM (2014). Physical activity interventions differentially affect exercise task and barrier self-efficacy: a meta-analysis. Health Psychol.

[47] Short CE, James EL, Vandelanotte C, Courneya KS, Duncan MJ, Rebar A, Plotnikoff RC (2014). Correlates of resistance training in post-treatment breast cancer survivors. Support Care Cancer.

[48] Australian Institute of Health Welfare (2022) Rural and remote health. AIHW, Canberra. https://www.aihw.gov.au/reports/rural-remote-australians/rural-and-remote-health

