# Supplementary material for: Self-efficacy, motivation, and habits: psychological correlates of exercise among women with breast cancer
Source: Support Care Cancer. 2023 Sep 20;31(10):584. doi: 10.1007/s00520-023-08040-7 (PMC10511352; doi:10.1007/s00520-023-08040-7)
Supplement: Supplementary file 1 — Supplementary file1 (DOCX 60 KB) [file 520_2023_8040_MOESM1_ESM.docx]

# Supplementary Materials

## **Supplementary Table 1.** Bivariate associations (n= 204)

|  | Total Activity  GLTEQ Total Activity Score | | | | | Resistance Training  Number of sessions per week | | | | |
| --- | --- | --- | --- | --- | --- | --- | --- | --- | --- | --- |
| Characteristics | **ꞵ** | **SE** | **95% Wald CI** | | ***P*** | **ꞵ** | **SE** | **95% Wald CI** | | ***P*** |
|  |  |  | **Lower** | **Upper** |  |  |  | **Lower** | **Upper** |  |
| Demographic Characteristics |  |  |  |  |  |  |  |  |  |  |
| Age | 0.04 | 0.13 | -0.22 | 0.30 | 0.76 | -0.02 | 0.01 | -0.04 | 0.00 | 0.10 |
| BMI | -1.02 | 0.24 | -1.50 | -0.54 | <0.001 | -0.03 | -0.02 | -0.07 | 0.02 | 0.22 |
| Comorbidity Index | -2.57 | 1.50 | -5.52 | 0.378 | 0.09 | -0.39 | 0.12 | -0.62 | -0.15 | 0.001 |
| Location |  |  |  |  | 0.56 |  |  |  |  | 0.83 |
| Major Cities Australia | Ref | Ref | Ref | Ref | Ref | Ref | Ref | Ref | Ref | Ref |
| Inner Regional/Outer Regional/Remote Australia | 1.74 | 2.99 | -4.12 | 7.60 | 0.56 | 0.05 | 0.25 | -0.43 | 0.54 | 0.83 |
| Marital Status (n=2 excluded, prefer not to say) |  |  |  |  | 0.60 |  |  |  |  | 0.93 |
| Married, de-facto, or living with partner | Ref | Ref | Ref | Ref | Ref | Ref | Ref | Ref | Ref | Ref |
| Separated, divorced, widowed, single | 1.67 | 3.20 | -4.60 | 7.93 | 0.60 | -0.02 | 0.27 | -0.55 | 0.50 | 0.93 |
| Education |  |  |  |  | 0.01 |  |  |  |  | 0.04 |
| High School (Year 10 or 12) | Ref | Ref | Ref | Ref | Ref | Ref | Ref | Ref | Ref | Ref |
| Certificate or Diploma (e.g., TAFE or College) | -7.27 | 5.73 | -18.50 | 3.97 | 0.21 | -0.61 | 0.47 | -1.54 | 0.31 | 0.20 |
| University Degree | 2.76 | 5.31 | -7.65 | 13.18 | 0.60 | 0.07 | 0.44 | -0.79 | 0.93 | 0.88 |
| Employment |  |  |  |  | 0.64 |  |  |  |  | 0.09 |
| Employed | Ref | Ref | Ref | Ref | Ref | Ref | Ref | Ref | Ref | Ref |
| Retired | 0.80 | 3.27 | -5.61 | 7.21 | 0.81 | -0.58 | 0.27 | -1.10 | -0.06 | 0.03 |
| Other | -3.06 | 3.90 | -10.70 | 4.58 | 0.43 | -0.31 | 0.32 | -0.93 | 0.31 | 0.32 |
| Disease Characteristics |  |  |  |  |  |  |  |  |  |  |
| Time Since Diagnosis | 0.45 | 0.92 | -1.36 | 2.26 | 0.63 | -0.05 | 0.08 | -0.20 | 0.09 | 0.48 |
| Stage |  |  |  |  | 0.01 |  |  |  |  | 0.91 |
| I-II | Ref | Ref | Ref | Ref | Ref | Ref | Ref | Ref | Ref | Ref |
| III-IV/Unsure | -8.26 | 3.14 | -14.41 | -2.11 | 0.01 | -0.03 | 0.26 | -0.54 | 0.48 | 0.91 |
| Treatment Type |  |  |  |  |  |  |  |  |  |  |
| No Surgery | Ref | Ref | Ref | Ref | Ref | Ref | Ref | Ref | Ref | Ref |
| Surgery | 3.91 | 7.41 | -10.62 | 18.43 | 0.60 | -0.05 | 0.61 | -1.24 | 1.14 | 0.93 |
| No Radiation | Ref | Ref | Ref | Ref | Ref | Ref | Ref | Ref | Ref | Ref |
| Radiation | -3.88 | 3.29 | -10.33 | 2.57 | 0.24 | 0.01 | 0.27 | -0.43 | 0.63 | 0.72 |
| No Chemotherapy | Ref | Ref | Ref | Ref | Ref | Ref | Ref | Ref | Ref | Ref |
| Chemotherapy | -2.13 | 2.89 | -7.79 | 3.52 | 0.46 | 0.22 | 0.24 | -0.24 | 0.69 | 0.34 |
| No Hormone Therapy | Ref | Ref | Ref | Ref | Ref | Ref | Ref | Ref | Ref | Ref |
| Hormone Therapy | -0.61 | 2.92 | -6.34 | 5.12 | 0.83 | -0.16 | 0.24 | -0.63 | 0.31 | 0.51 |
| No Herceptin | Ref | Ref | Ref | Ref | Ref | Ref | Ref | Ref | Ref | Ref |
| Herceptin | -7.52 | 4.21 | -15.78 | 0.74 | 0.07 | 0.41 | 0.35 | -0.27 | 1.09 | 0.24 |
| No Immunotherapy | Ref | Ref | Ref | Ref | Ref | Ref | Ref | Ref | Ref | Ref |
| Immunotherapy | 1.12 | 7.01 | -12.62 | 14.85 | 0.87 | 0.80 | 0.57 | -0.33 | 1.91 | 0.16 |
| No Other Treatment | Ref | Ref | Ref | Ref | Ref | Ref | Ref | Ref | Ref | Ref |
| Other Treatment | -10.02 | 7.38 | -24.49 | 4.45 | 0.18 | 0.05 | 0.61 | -1.14 | 1.24 | 0.93 |
| Treatment Stage (n=5 excluded, not yet started + other) |  |  |  |  | 0.28 |  |  |  |  | 0.11 |
| Current undergoing curative treatment | Ref | Ref | Ref | Ref | Ref | Ref | Ref | Ref | Ref | Ref |
| Completed curative treatment and in remission | -0.85 | 3.88 | -8.45 | 6.75 | 0.83 | -0.45 | 0.31 | -1.06 | 0.17 | 0.16 |
| Ongoing treatment to manage the disease | -6.18 | 4.56 | -15.13 | 2.76 | 0.18 | -0.78 | 0.37 | -1.51 | -0.06 | 0.03 |
| Psychological Constructs |  |  |  |  |  |  |  |  |  |  |
| Self-Efficacy |  |  |  |  |  |  |  |  |  |  |
| Task SE Score | 0.19 | 0.07 | 0.04 | 0.33 | 0.010 | 0.03 | 0.01 | 0.01 | 0.04 | <0.001 |
| Coping SE Score | 0.24 | 0.07 | 0.10 | 0.37 | <0.001 | 0.02 | 0.01 | 0.01 | 0.04 | <0.001 |
| Scheduling SE Score | 0.37 | 0.05 | 0.27 | 0.47 | <0.001 | 0.03 | 0.00 | 0.02 | 0.04 | <0.001 |
| Motivation |  |  |  |  |  |  |  |  |  |  |
| Amotivation | -10.62 | 3.04 | -16.58 | -4.65 | <0.001 | -0.52 | 0.25 | -1.02 | -0.02 | 0.04 |
| External Motivation | -5.10 | 2.08 | -9.19 | -1.02 | 0.01 | -0.19 | 0.17 | -0.53 | 0.15 | 0.27 |
| Introjected | -2.23 | 1.37 | -4.91 | 0.45 | 0.10 | -0.08 | 0.11 | -0.30 | 0.14 | 0.49 |
| Identified | 11.38 | 1.65 | 8.15 | 14.61 | <0.001 | 0.76 | 0.14 | 0.49 | 1.04 | <0.001 |
| Intrinsic | 6.66 | 1.25 | 4.20 | 9.12 | <0.001 | 0.49 | 0.10 | 0.29 | 0.69 | <0.001 |
| Habits |  |  |  |  |  |  |  |  |  |  |
| Automaticity of PA (Mean) | 8.07 | 1.41 | 5.32 | 10.83 | <0.001 | 0.48 | 0.12 | 0.24 | 0.71 | <0.001 |

## **Supplementary Table 2.** Correlation Matrix for Independent Variables

|  | Age | Body Mass Index | Education Status | Stage of Disease | Comorbidity Index | Task Self-efficacy | Coping Self-efficacy | Scheduling Self-efficacy | Amotivation | External Motivation | Identified  Motivation | Intrinsic  Motivation | Habits |
| --- | --- | --- | --- | --- | --- | --- | --- | --- | --- | --- | --- | --- | --- |
| Age | -- |  |  |  |  |  |  |  |  |  |  |  |  |
| Body Mass Index | -0.04 | -- |  |  |  |  |  |  |  |  |  |  |  |
| Education Status | -0.20 | <-0.01 | -- |  |  |  |  |  |  |  |  |  |  |
| Stage of Disease | -0.06 | 0.03 | 0.02 | -- |  |  |  |  |  |  |  |  |  |
| Comorbidity Index | 0.22 | 0.28 | -0.17 | 0.03 | -- |  |  |  |  |  |  |  |  |
| Task Self-efficacy | -0.04 | -0.11 | 0.07 | <-0.01 | -0.23 | -- |  |  |  |  |  |  |  |
| Coping Self-efficacy | -0.12 | -0.13 | 0.09 | 0.09 | -0.27 | 0.47 | -- |  |  |  |  |  |  |
| Scheduling Self-efficacy | 0.07 | -0.26 | 0.14 | 0.02 | -0.23 | 0.55 | 0.45 | -- |  |  |  |  |  |
| Amotivation | <0.01 | 0.11 | -0.25 | 0.09 | 0.02 | -0.25 | -0.08 | -0.34 | -- |  |  |  |  |
| External Motivation | -0.18 | 0.23 | <-0.01 | 0.06 | 0.10 | -0.10 | -0.05 | -0.20 | 0.29 | -- |  |  |  |
| Identified Motivation | -0.08 | -0.32 | 0.25 | -0.03 | -0.19 | 0.47 | 0.35 | 0.68 | -0.53 | -0.19 | -- |  |  |
| Intrinsic Motivation | -0.08 | -0.23 | 0.13 | -0.01 | -0.18 | 0.51 | 0.38 | 0.62 | -0.40 | -0.28 | 0.73 | -- |  |
| Habits | 0.09 | -0.23 | -0.03 | <-0.01 | -0.22 | 0.38 | 0.20 | 0.57 | -0.19 | -0.22 | 0.47 | 0.56 | -- |
